# Supplementary material for: Socioeconomic determinants of hypertension and prehypertension in Peru: Evidence from the Peruvian Demographic and Health Survey
Source: PLoS One. 2021 Jan 26;16(1):e0245730. doi: 10.1371/journal.pone.0245730 (PMC7837486; doi:10.1371/journal.pone.0245730)
Supplement: S1 Fig — (DOCX) [file pone.0245730.s001.docx]

**S1 Fig. Awareness of hypertension by area of residence, ENDES 2018**
